# Supplementary material for: Network and pathway expansion of genetic disease associations identifies successful drug targets
Source: Sci Rep. 2020 Dec 1;10:20970. doi: 10.1038/s41598-020-77847-9 (PMC7708424; doi:10.1038/s41598-020-77847-9)
Supplement: Supplementary file 1 — Supplementary Information 1. [file 41598_2020_77847_MOESM1_ESM.docx]

# Network and pathway expansion of genetic disease associations identifies successful drug targets

## Authors

Aidan MacNamara: aidan.macnamara@gmail.com, Functional Genomics, GSK, Stevenage, UK

Nikolina Nakic: nikolina.x.nakic@gsk.com, Functional Genomics, GSK, Stevenage, UK

Ali Amin: ali.x.amin@gsk.com, Human Genetics, GSK, Stevenage, UK

Cong Guo: karl.x.guo@gsk.com, Human Genetics, GSK, Collegeville PA

Karsten B. Sieber: karsten.b.sieber@gsk.com, Human Genetics, GSK, Collegeville PA

Mark Hurle: mark.r.hurle@gsk.com, Human Genetics, GSK, Collegeville PA

Alex Gutteridge (contact): alex.x.gutteridge@gsk.com, Functional Genomics, GSK, Stevenage, UK


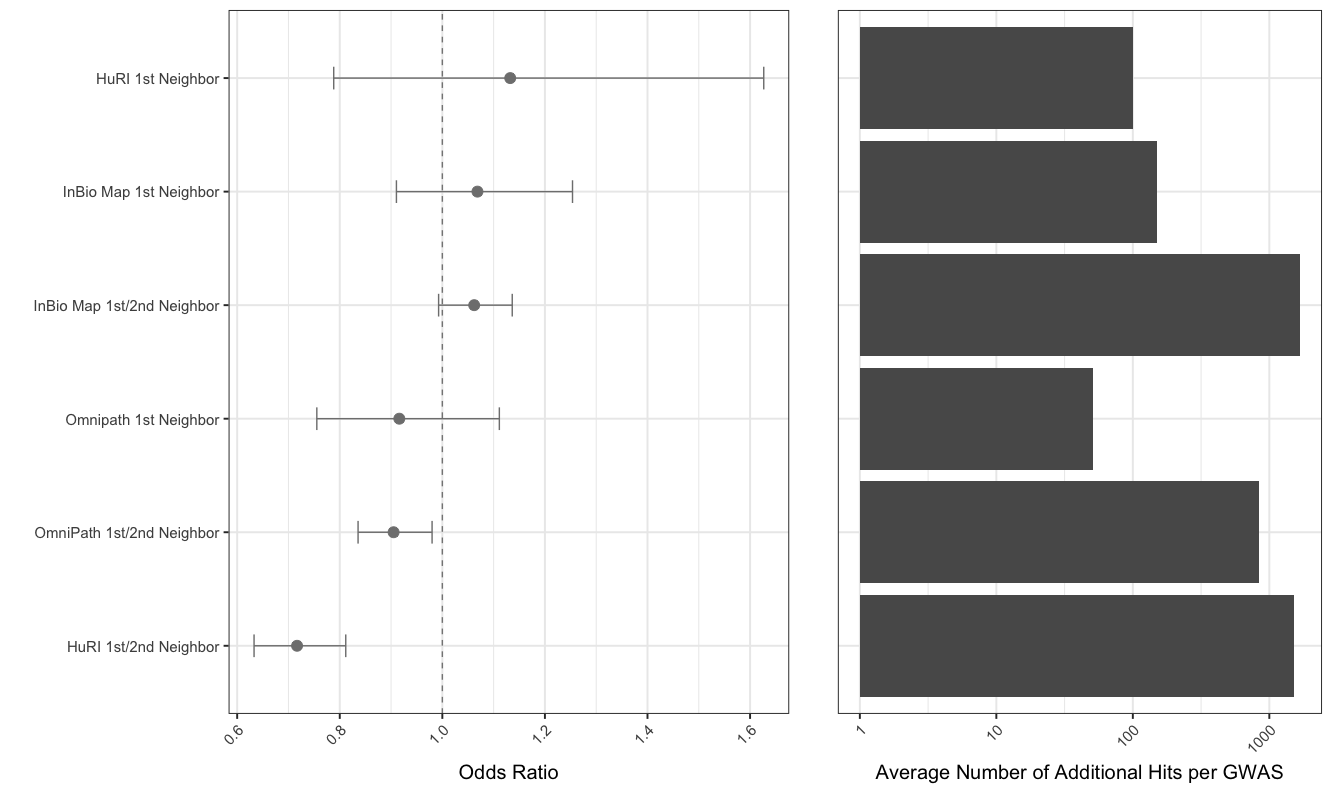


Supplementary Figure 1: Enrichment of successful drug targets (left) and number of targets implicated (right) using the naïve approach of first and second neighbors across different network sources (HuRI, InBio Map, STRING, OmniPath)


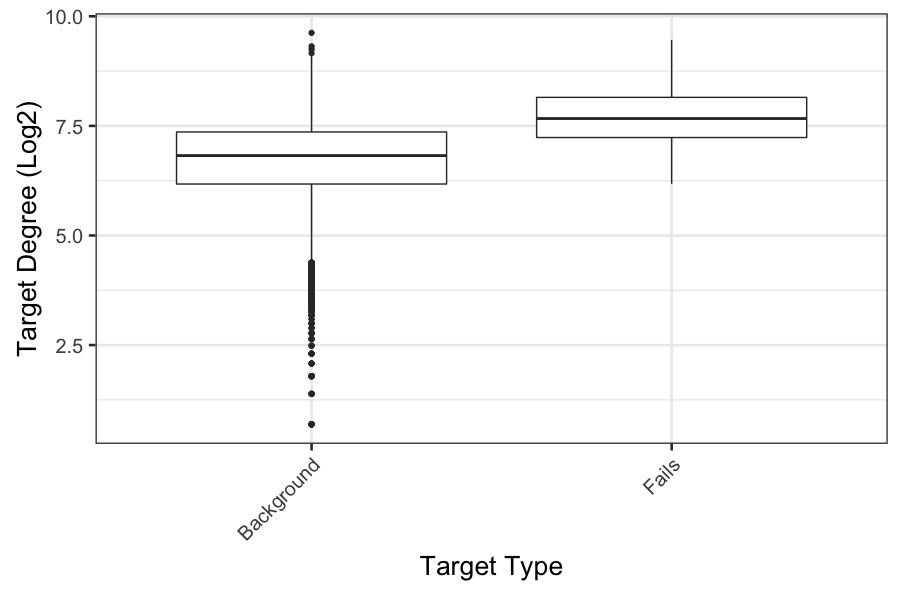


Supplementary Figure 2: A comparison of the node degree (the number of connections in a network that a gene has) between genes that have the most clinical failures *(n = 500)* against all other genes.

| **Method** | **Odds** | **Confidence Intervals 95%** | | **P** |
| --- | --- | --- | --- | --- |
| HCGH | 3.77 | 2.82 | 5.06 | 0.0000 |
| Complex | 3.68 | 1.68 | 8.05 | 0.0014 |
| Pascal Co-Expresssion | 3.09 | 1.59 | 5.99 | 0.0003 |
| Ligand Receptor | 2.00 | 1.67 | 2.41 | 0.0000 |
| Pascal PPI | 1.92 | 1.16 | 3.16 | 0.0088 |
| HotNet2 Inbio Map | 1.88 | 1.47 | 2.42 | 0.0000 |
| Pascal Metabase | 1.78 | 1.35 | 2.34 | 0.0000 |
| HotNet2 OmniPath | 1.37 | 1.11 | 1.69 | 0.0035 |
| Pascal Reactome | 1.35 | 0.99 | 1.84 | 0.0587 |
| Pathway 1^st^ Neighbor | 1.26 | 0.98 | 1.62 | 0.0728 |
| HotNet2 STRING | 1.22 | 0.79 | 1.88 | 0.4206 |
| HuRI 1^st^ Neighbor | 1.13 | 0.79 | 1.63 | 0.5248 |
| InBio Map 1^st^ Neighbor | 1.07 | 0.91 | 1.25 | 0.4200 |
| InBio Map 1^st^/2^nd^ Neighbor | 1.06 | 0.99 | 1.14 | 0.0791 |
| HotNet2 HuRI | 1.01 | 0.71 | 1.44 | 0.9386 |
| Random | 1.01 | 0.96 | 1.06 | 0.8275 |
| STRING 1^st^ Neighbor | 0.98 | 0.90 | 1.05 | 0.5227 |
| OmniPath 1^st^ Neighbor | 0.92 | 0.76 | 1.11 | 0.3888 |
| OmniPath 1^st^/2^nd^ Neighbor | 0.90 | 0.84 | 0.98 | 0.0150 |
| Pathway 1^st^/2^nd^ Neighbor | 0.72 | 0.63 | 0.83 | 0.0000 |
| HuRI 1^st^/2^nd^ Neighbor | 0.72 | 0.63 | 0.81 | 0.0000 |
| All Pathway | 0.71 | 0.66 | 0.76 | 0.0000 |
| STRING 1^st^/2^nd^ Neighbor | 0.49 | 0.42 | 0.56 | 0.0000 |

Supplementary Table 1: The odds ratio and 95% confidence intervals of successful drug enrichment across all methods


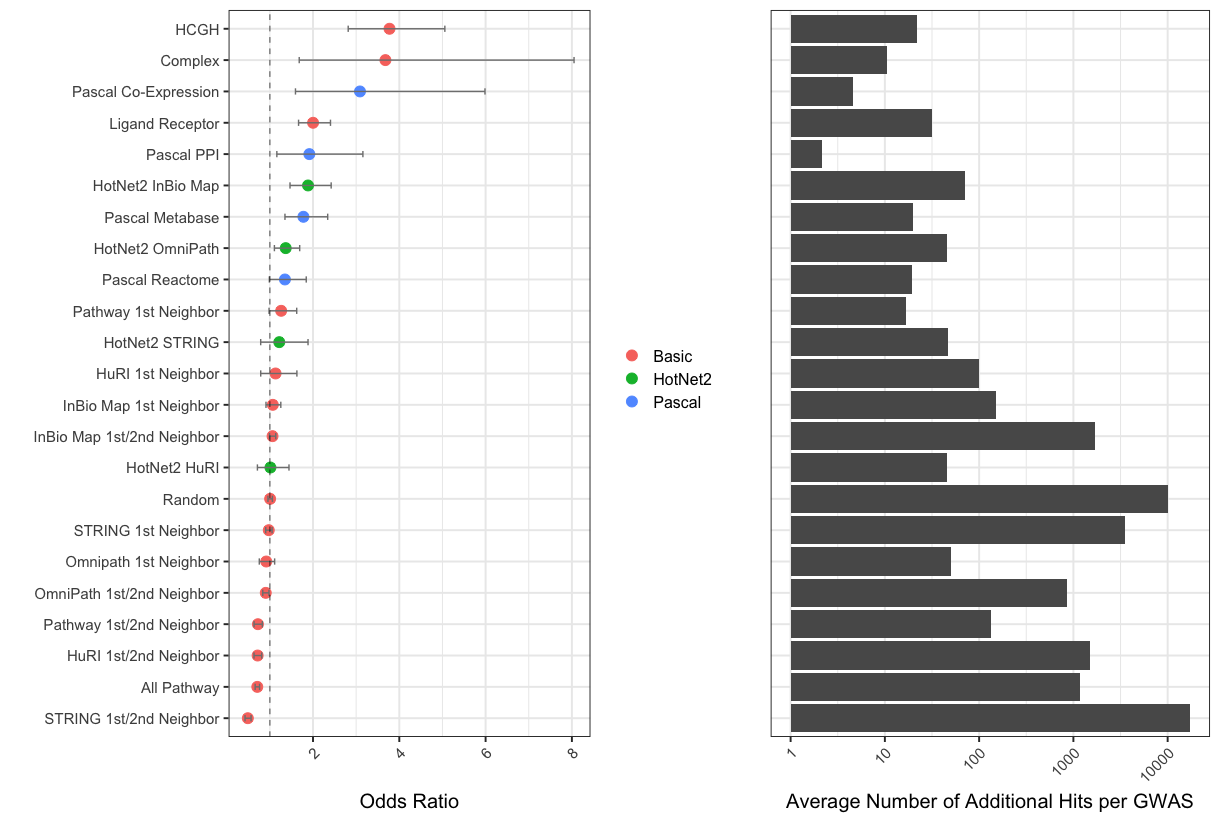


Supplementary Figure 3: Enrichment of successful drug targets (left) and number of targets implicated (right) across all methods.


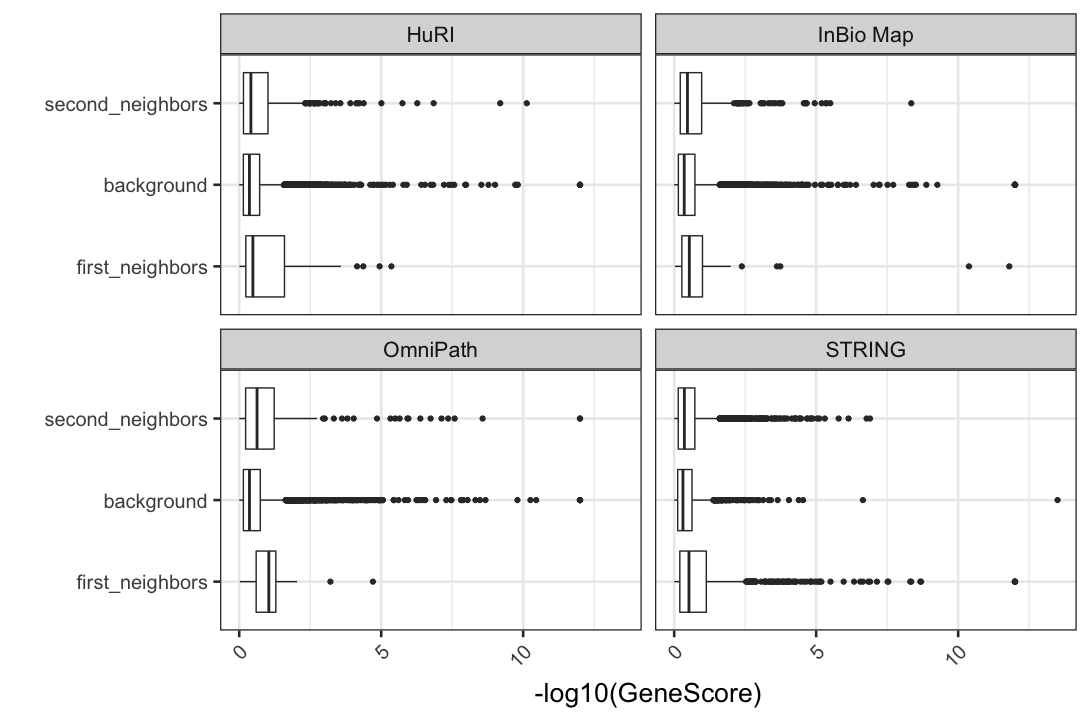


Supplementary Figure 4: A comparison of the Pascal gene score across the 4 networks for first and second neighbors of HCGHs.


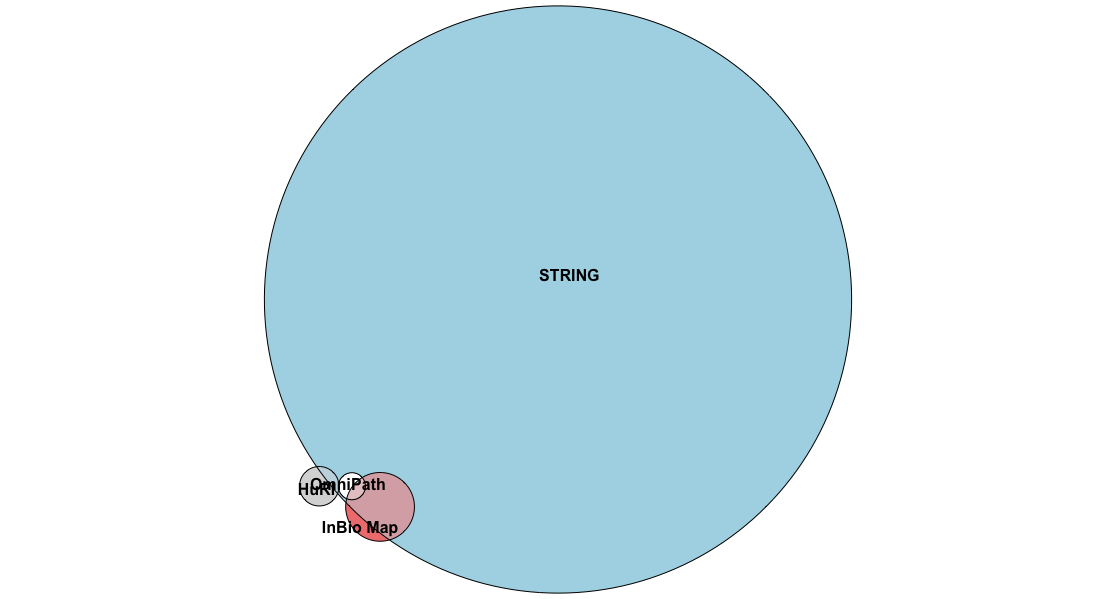


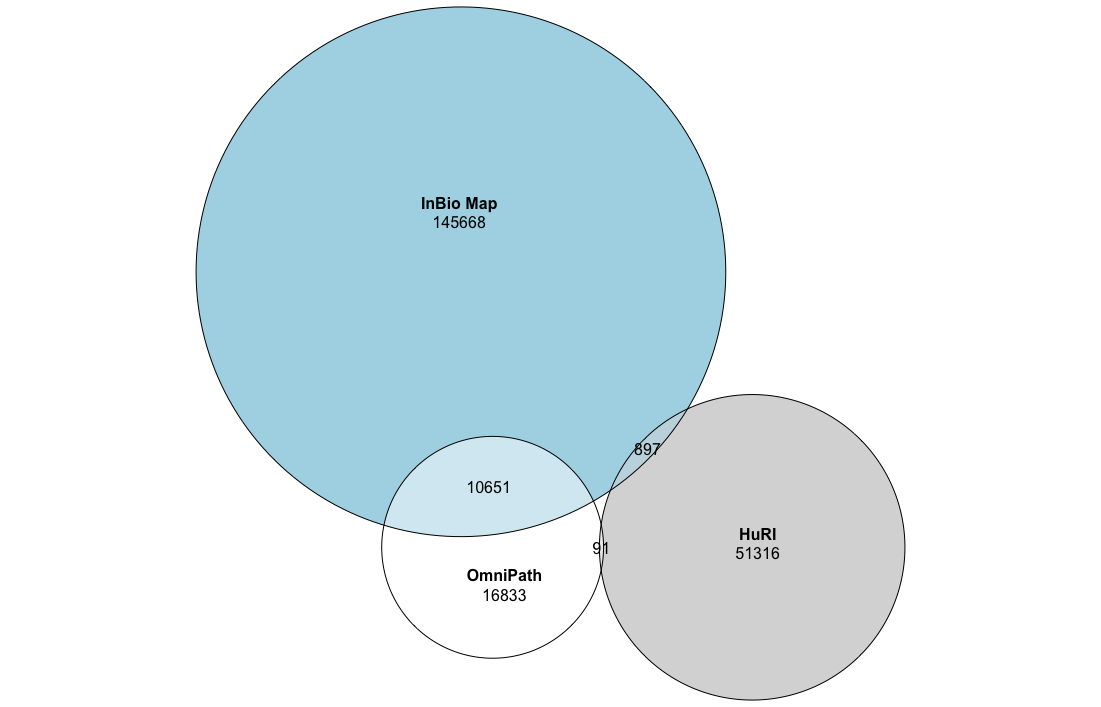


Supplementary Figure 5: The overlap in unique interactions across the different network resources used.


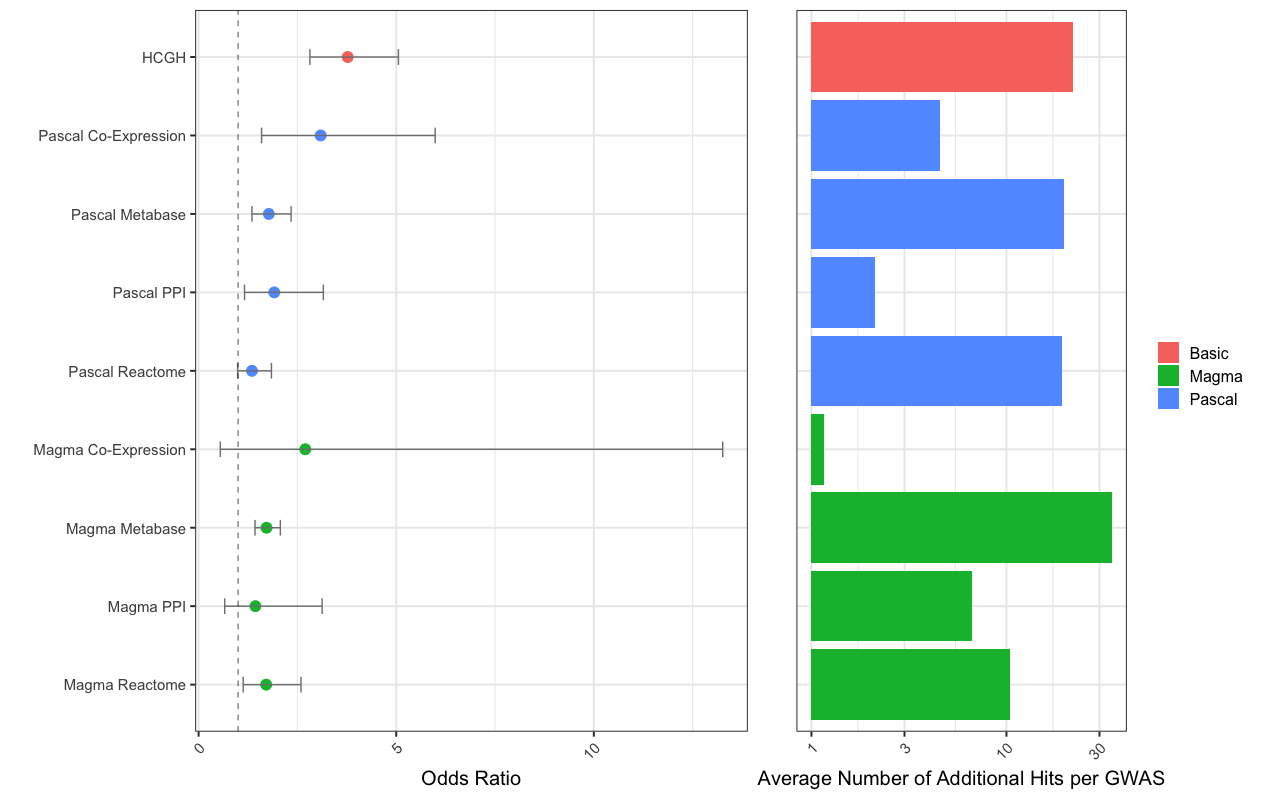


Supplementary Figure 6: Enrichment of successful drug targets (left) and number of targets implicated (right) for Pascal and Magma (with the HCGH reference)
